# Spontaneous Rupture of Hepatic Metastasis from Pancreatic Adenocarcinoma

**DOI:** 10.1155/2016/6968534

**Published:** 2016-08-15

**Authors:** Anil Rahul, Fernandes Robin, Hiremath Adarsh

**Affiliations:** Internal Medicine, MD Anderson Cancer Center, 1400 Pressler Street, Houston, TX 77030, USA

## Abstract

A 58-year-old man with advanced-stage pancreatic adenocarcinoma presented with fatigue and dyspnea. Examination revealed tachycardia (102 b/min) with mild tenderness in right upper quadrant. His hemoglobin (Hb) was 7.9 g/dL (10 days prior to presentation 12.2 g/dL), International normalized ratio (INR), platelet count was normal, and the stool guaiac test was negative. On admission, abdominal computed tomography (CT) scan showed hepatic metastatic lesion with a rupture and hemoperitoneum communicating to the subdiaphragmatic space. This rapid progression of anemia along with presenting symptoms and CT imaging were attributed to diagnosis of spontaneous rupture of liver metastasis from pancreatic adenocarcinoma. Patient received blood transfusion and hemoglobin was monitored in successive intervals. His general condition and anemia improved with conservative management and he was discharged in 3 days. Repeated CT after 4 months showed resolving hemoperitoneum and stable hemoglobin levels. The patient deceased 9 months after being diagnosed. A literature search revealed limited data regarding the incidence and management of spontaneous rupture of metastatic lesion secondary to pancreatic adenocarcinoma which has been managed conservatively and thus we are reporting our experience.

## 1. Introduction

Spontaneous ruptures secondary to malignant liver metastasis are uncommon occurrences that clinically present with similar symptoms/signs as the more common spontaneous rupture of liver secondary to hepatocellular carcinoma (HCC). HCC is consistently reported to have a poor prognosis and, due to its prevalence, continues to be the most lethal and life-threatening hallmark of advanced disease, attributing to 25–75% mortality in the acute phase HCC [1]. Hepatic metastatic rupture, on the other hand, has a comparably poorer prognosis owing to confounding clinical presentation and delayed diagnosis. Several invasive interventional methods have been developed to prevent fatal sequelae of intraperitoneal hemorrhage. Thus, these invasive interventions have become the most prevalent methods of treatment, far surpassing conservative therapies. However, the interventional strategies do not significantly improve median survival and result in recurrent complications and interruptions to chemotherapy. Herein, we present a rare case of successful conservative management of spontaneous rupture of liver metastasis secondary to advanced pancreatic adenocarcinoma with no delay in resuming immunotherapy for cancer.

## 2. Case Report

**Figure 1:**
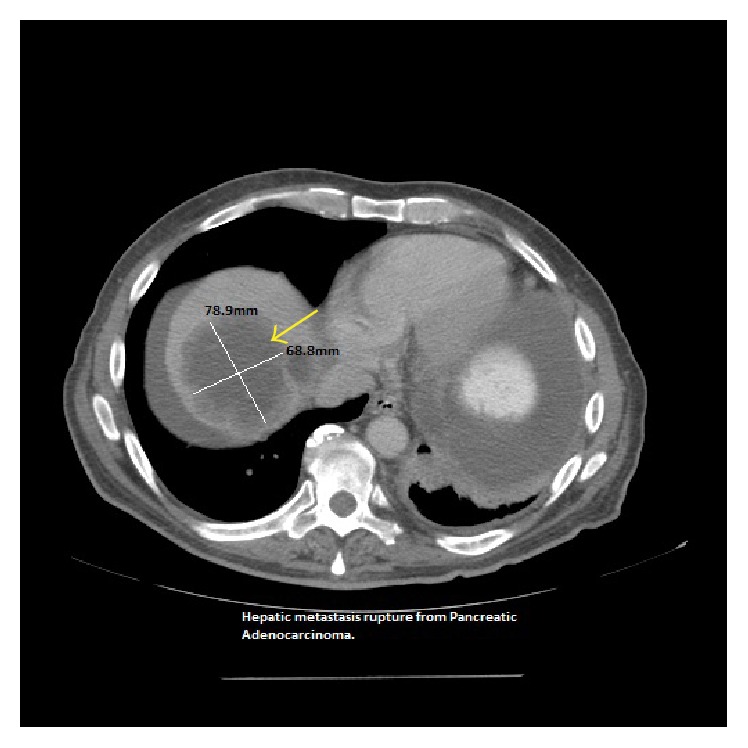
Axial contrast enhanced CT image showing metastasis in liver dome (large yellow arrow) had grown since earlier study (Figure 2).

**Figure 2:**
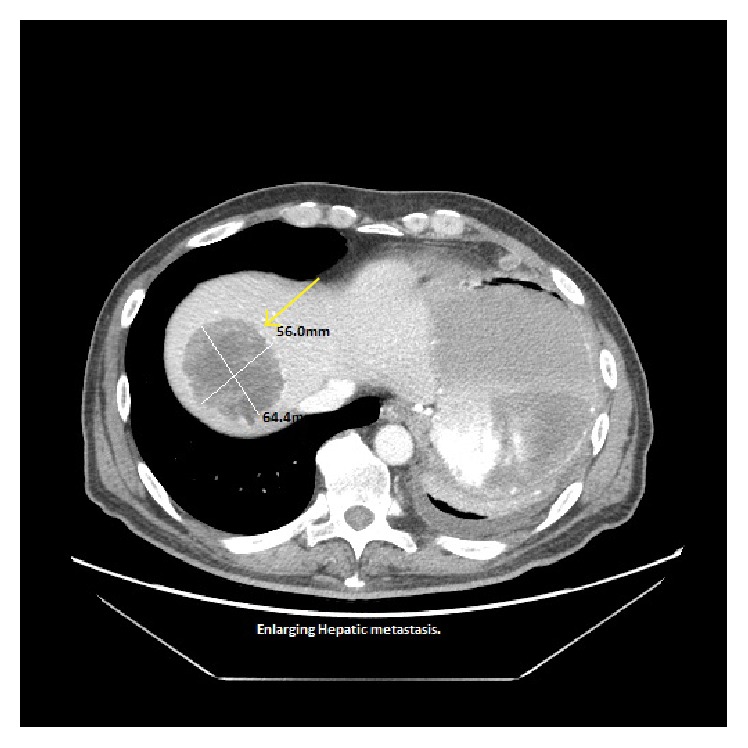
Previous CT (contrast enhanced) showing liver metastases (yellow arrow) smaller at that time and no perihepatic fluid.

**Figure 3:**
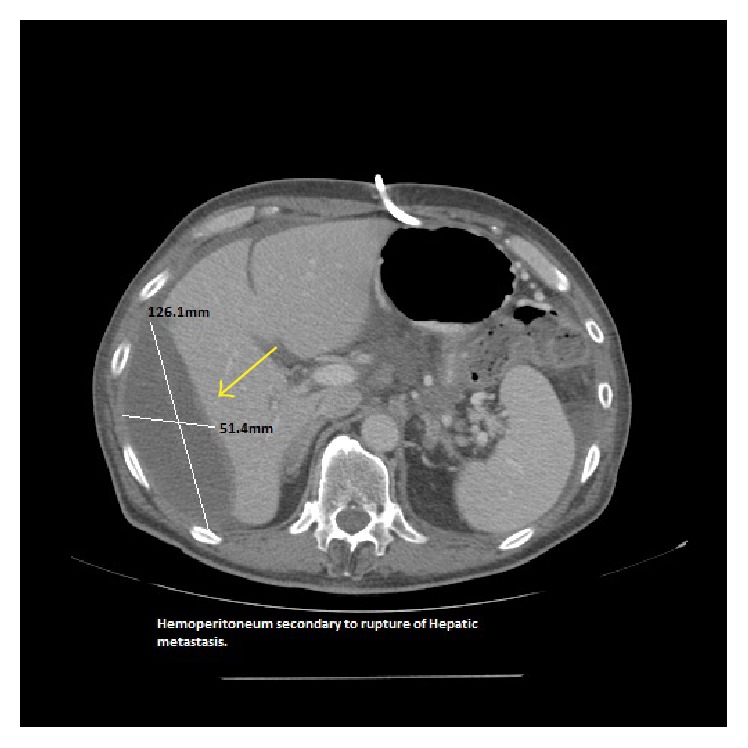
Axial contrast enhanced CT image (same study as in Figure 1) showing (yellow arrow) subcapsular liver collection.

**Figure 4:**
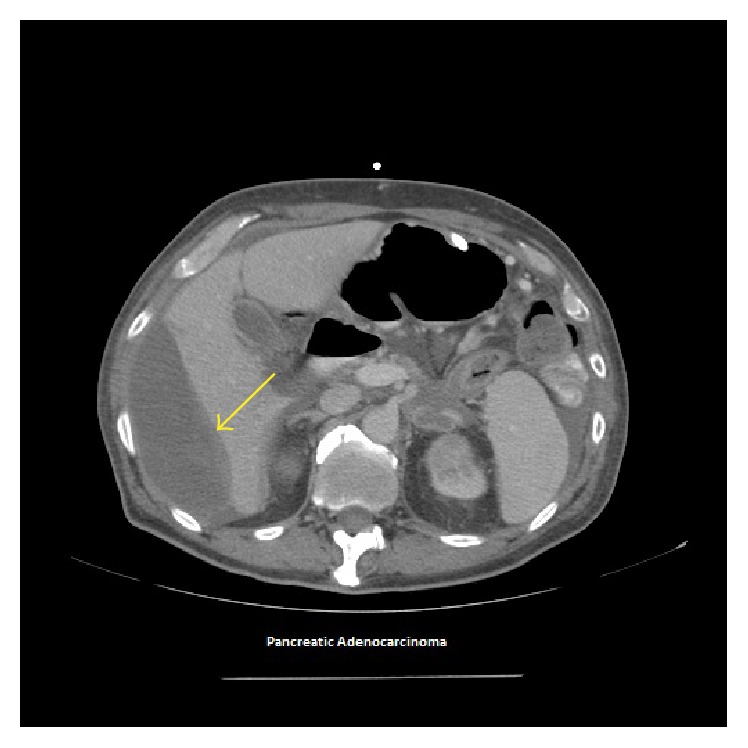
Axial contrast enhanced CT obtained 4 months after study in Figures 1 and 3 showing decreasing size of subcapsular liver collection.

A 58-year-old man presented with a 2-week history of fatigue and dyspnea. Two years prior to the presentation, he was diagnosed with pancreatic adenocarcinoma in the body and tail which subsequently metastasized to the liver, left lung, 6th cervical vertebra (c-6), splenic artery, ascitic fluid, and diaphragm which was confirmed by biopsies at aforementioned sites. In the interim, he underwent several lines of chemotherapy, radiation, and molecularly targeted therapy. Surgical procedures included wedge resection of the left lung lesions, cervical corpectomy and fusion, abdominal paracentesis, and gastrostomy tube placement. Past medical history includes type 2 diabetes mellitus and tubulovillous adenoma for which he underwent polypectomy. On admission, the patient was in minimal distress with tachycardia (102 beats/min), blood pressure of 99/62 mmHg, and mild tenderness in the right upper quadrant of abdomen. Laboratory data revealed hemoglobin (Hb): 7.9 g/dL; hematocrit (Hct): 24.9%; platelet count: 210,000/mm^3^; prothrombin time (PT): 14 s; INR: 1.08; activated partial thromboplastin time: 27.7 s; stool guaiac test that was negative; LFT's and RFT's that were within normal limits. 10 days prior to this presentation, his Hb and Hct levels were 12.2 g/dL and 37.7%, respectively, suggesting rapid progression of anemia. Abdominal contrast enhanced CT was immediately performed and revealed an enlarging hepatic metastatic lesion (78.9 × 68.8 mm) with a rupture (Figure 1) and hemoperitoneum (126.1 × 51.4 mm) with communication to the subdiaphragmatic space (Figure 3). Previous CT imaging showed a hepatic metastatic lesion measuring (56.0 × 64.4 mm) (Figure 2). Follow-up imaging revealed resolving hemoperitoneum (Figure 4).

Considering the guarded condition and vitals being the lower limits of normalcy, the medical team consisting of a hospitalist, interventional radiologist, and oncologist decided upon managing the patient's treatment conservatively. The patient was given a blood transfusion; Hb and Hct were monitored serially in successive intervals during hospitalization. Subsequently, his general condition and anemia improved (Hb to 9.9 g/dL and Hct to 30.9%). Throughout his hospitalization, the patient's condition was stable and improved solely with conservative management. Since this approach was undertaken, there was no undue delay in resuming the patient's immunotherapy for pancreatic cancer. Repeated CT after 4 months showed resolving hemoperitoneum and stable Hb. The patient deceased 9 months after being diagnosed with hemoperitoneum secondary to metastatic hepatic rupture.

## 3. Discussion

Pancreatic cancer is one of the deadliest cancers, evidenced by the fact that its mortality equals its incidence [2]. Early diagnosis and treatment are limited for this condition because of the tumor's aggressively invasive nature and early metastatic properties. Pancreatic cancer metastasizes early in its course and liver is the most common site for distant metastasis, followed by the peritoneal cavity [3]. Massive hemorrhage related to ruptured liver metastases is quite exceptional and less than 50 cases are reported in the literature. Spontaneous rupture of a metastatic liver tumor is rare and uncommon when compared to hepatic rupture of primary lesion (HCC) leading to hemoperitoneum, which is a devastating complication of both primary and metastatic hepatic tumors [4, 5].

Pivotal clinical features of hemoperitoneum indicating hepatic metastatic rupture include history of malignancy, abdominal pain, hypotension, severe anemia, elevated liver enzymes, and, in extreme cases, surgical abdomen. Choi et al. reported that peripheral location of the tumor, protruding, contour, discontinuity of hepatic surface, and surrounding hemoperitoneum are helpful diagnostic indicators of ruptured HCC [6]. The aforementioned clinical and imaging (CT) findings helped us to yield to a diagnosis of metastatic hepatic rupture in our patient.

Treatment of hemoperitoneum secondary to spontaneous rupture of metastatic liver tumor depends on several factors, including tumor size, location, and severity of exsanguination. The major objective of treatment is to control the hemorrhage quickly and effectively; this can be accomplished by hepatic wedge resection/lobectomy or suture ligation of the bleeding source/hepatic artery. Recent studies suggested that a two-staged therapeutic approach in managing ruptured hepatic lesion consists of initial management with conservative approach, hemostasis achieved via transarterial embolization or surgery, followed by staged hepatic resection [7]. Transcatheter hepatic arterial embolization (TAE) may seem to be ideal for these patients, due to its various merits. Greatest benefit of TAE would be being done under local anesthesia and less invasive nature of the procedure [8]. That said, it also has some demerits, like recurrent bleeding and liver failure [8, 9], peritoneal abscess [10], implanted metastases, and patients who need to have preserved liver functions (not beyond Child-Pugh B) [11]. In addition, long-term results are poor if it is used as a solitary treatment approach without being followed by surgery [12]. On the other hand, even though surgery showed better mortality benefit [13], it has numerous risk like infections, and secondary bleeding, high morbidity [14], and last but not the least it puts a far greater toll on inveitable interruptions in chemo/immunotherapies, increase in hospital stay and cost.

However, conservative treatment focuses on achieving hemostatsis by correcting coagulopathy, close monitoring, and follow-up medical imaging to confirm hemostasis after initial resuscitation [9]. A study by Hsueh et al. showed patients who received hepatectomy, either immediate or staged after posttransarterial embolization, and reported higher survival rates of 85.2% at 30 days and 62.2% at 1 year. By comparison, similar populations treated conservatively exhibited a reduction in liver function, prolonged INR, and increased 30-day mortality [15]. Contrary to the above study Leung et al. in their retrospective study on 112 patients with ruptured HCC, comparing the in-hospital mortality and median survival in patients treated with conservative and surgical approach, concluded that the conservative approach gave similar results to that of surgical approach [(62% versus 51%) and (7 days' versus 12 days) resp.] [16]. Having arrived at the diagnosis, with consideration of the risk factors in this patient, we agreed on managing the patient's treatment conservatively despite the odds of poor prognosis, and he responded very well and was discharged home in stable condition to resume immunotherapy for pancreatic cancer without any interruptions.

## 4. Conclusion

In conclusion, we convey that spontaneous metastatic liver rupture secondary to advanced pancreatic adenocarcinoma is rare and has a poor prognosis. Clinical findings and CT are of great help in making a quick diagnosis of hemoperitoneum due to metastatic liver rupture. Although the literature supports invasive intervention (extrapolating from data for HCC) in advanced-stage pancreatic cancer, conservative treatment alone (if medically stable) can be employed to combat the sequelae of intraperitoneal hemorrhage and improve survival with fewer complications and minimal interruption or delays in necessary chemotherapy or cancer-directed treatments.

## References

[1] Aoki T., Kokudo N., Matsuyama Y. (2014). Prognostic impact of spontaneous tumor rupture in patients with hepatocellular carcinoma: an analysis of 1160 cases from a nationwide survey. *Annals of Surgery*.

[2] Siegel R., Ma J., Zou Z., Jemal A. (2014). Cancer statistics, 2014. *CA Cancer Journal for Clinicians*.

[3] Yachida S., Jones S., Bozic I. (2010). Distant metastasis occurs late during the genetic evolution of pancreatic cancer. *Nature*.

[4] Tung C.-F., Chang C.-S., Chow W.-K., Peng Y.-C., Hwang J.-I., Wen M.-C. (2002). Hemoperitoneum secondary to spontaneous rupture of metastatic epidermoid carcinoma of liver: case report and review of the literature. *Hepato-Gastroenterology*.

[5] Chen Z.-Y., Qi Q.-H., Dong Z.-L. (2002). Etiology and management of hemmorrhage in spontaneous liver rupture: a report of 70 cases. *World Journal of Gastroenterology*.

[6] Choi B. G., Park S. H., Byun J. Y., Jung S. E., Choi K. H., Han J.-Y. (2001). The findings of ruptured hepatocellular carcinoma on helical CT. *The British Journal of Radiology*.

[7] Veltchev L. M. (2009). Spontaneous rupture of hepatocellular carcinoma and hemoperitoneum-management and long term survival. *Journal of IMAB*.

[8] Leung C. S., Tang C. N., Fung K. H., Li M. K. W. (2002). A retrospective review of transcatheter hepatic arterial embolisation for ruptured hepatocellular carcinoma. *Journal of the Royal College of Surgeons of Edinburgh*.

[9] Tanaka A., Takeda R., Mukaihara S. (2001). Treatment of ruptured hepatocellular carcinoma. *International Journal of Clinical Oncology*.

[10] Yokoi Y., Suzuki S., Sakaguchi T. (2002). Subphrenic abscess formation following superselective transcatheter chemoembolization for hepatocellular carcinoma. *Radiation Medicine*.

[11] Yeh C.-N., Chen H.-M., Chen M.-F., Chao T.-C. (2002). Peritoneal implanted hepatocellular carcinoma with rupture after TACE presented as acute appendicitis. *Hepato-Gastroenterology*.

[12] Rossetto A., Adani G. L., Risaliti A. (2010). Combined approach for spontaneous rupture of hepatocellular carcinoma. *World Journal of Hepatology*.

[13] Jin Y.-J., Lee J.-W., Park S.-W. (2013). Survival outcome of patients with spontaneously ruptured hepatocellular carcinoma treated surgically or by transarterial embolization. *World Journal of Gastroenterology*.

[14] Chen W.-K., Chang Y.-T., Chung Y.-T., Yang H.-R. (2005). Outcomes of emergency treatment in ruptured hepatocellular carcinoma in the ED. *The American Journal of Emergency Medicine*.

[15] Hsueh K.-C., Fan H.-L., Chen T.-W. (2012). Management of spontaneously ruptured hepatocellular carcinoma and hemoperitoneum manifested as acute abdomen in the emergency room. *World Journal of Surgery*.

[16] Leung K. L., Lau W. Y., Lai P. B. S., Yiu R. Y. C., Meng W. C. S., Leow C. K. (1999). Spontaneous rupture of hepatocellular carcinoma: conservative management and selective intervention. *Archives of Surgery*.

